# Decreased Frequencies of Th17 and Tc17 Cells in Patients Infected with Avian Influenza A (H7N9) Virus

**DOI:** 10.1155/2019/1418251

**Published:** 2019-04-02

**Authors:** Jiaqi Bao, Dawei Cui, Xiaochen Wang, Qianda Zou, Dejian Zhao, Shufa Zheng, Fei Yu, Li Huang, Yuejiao Dong, Xianzhi Yang, Guoliang Xie, Weizhen Chen, Yu Chen

**Affiliations:** ^1^Center of Clinical Laboratory, The First Affiliated Hospital, Zhejiang University School of Medicine, Hangzhou, Zhejiang 310003, China; ^2^Key Laboratory of Clinical In Vitro Diagnostic Techniques of Zhejiang Province, Hangzhou, Zhejiang 310002, China; ^3^State Key Laboratory for Diagnosis and Treatment of Infectious Diseases, Collaborative Innovation Center for Diagnosis and Treatment of Infectious Diseases, The First Affiliated Hospital, Zhejiang University School of Medicine, Hangzhou, Zhejiang 310003, China

## Abstract

The outbreak of avian influenza A (H7N9) virus infection, with a high mortality rate, has caused concern worldwide. Although interleukin-17- (IL-17-) secreting CD4^+^ T (Th17) and CD8^+^ T (Tc17) cells have been proven to play crucial roles in influenza virus infection, the changes and roles of Th17 and Tc17 cells in immune responses to H7N9 infection remain controversial. In this study, we found that the frequencies of Th17 and Tc17 cells among human peripheral blood mononuclear cells (PBMCs) as well as IL-17A protein and mRNA levels were markedly decreased in patients with acute H7N9 virus infection. A positive correlation was found between the serum IL-17A level and the frequency of these two cell groups. *In vitro* infection experiments revealed decreased Th17 and Tc17 cell frequency and IL-17A levels at various time points postinfection. In addition, Th17 cells were the predominant sources of IL-17A in PBMCs of patients infected with H7N9 virus. Taken together, our results indicate immune disorder in acute H7N9 infection and a restored Th17 and Tc17 cell frequency might serve as a biomarker for predicting recovery in patients infected with this virus.

## 1. Introduction

A novel and highly virulent avian influenza A (H7N9) virus associated with human death but no apparent death in poultry and wild birds has emerged in eastern China since March 2013 [1]. Avian influenza A (H7N9) virus is a novel influenza A virus with a genome consisting of eight segments of negative-sense single-stranded RNA [2]. Unlike other influenza A virus subtypes, H7N9 infections are more severe, and cases of infection with this virus are generally characterized by acute community-acquired pneumonia that rapidly develops into acute respiratory distress syndrome (ARDS), multiorgan dysfunction (MOD), shock, and even death [3–5]. To date, there have been five H7N9 infection waves in China [6, 7], with 1,564 laboratory-confirmed cases and at least 612 deaths, which constitutes an ongoing public health threat [8].

Several studies have investigated the changes in immune cell subsets and cytokine profiles of patients with H7N9 infection. For example, Huang et al. reported elevated levels of cytokines and antibodies in serum samples of H7N9 patients with acute infection [9]. Chen et al. demonstrated that the levels of T cell subsets were lower in critically ill patients than in patients who recovered from H7N9 infection [10], and Diao et al. found patients with severe infection to be lymphopenic, with significantly decreased CD14^+^ cell antigen-presenting capacity and levels of related cytokines [11]. Despite the distinct features of H7N9 infection, detailed knowledge of the immune status and immune patterns in these patients remains limited.

Adaptive cell immunity plays a pivotal role in the response to influenza A virus infections, and T cell-mediated immune responses during H7N9 virus infection have been reported to indicate host immune pathogenesis or protection mechanisms [12]. Novel T cell subsets such as Th17 cells [13] and Tc17 cells [14] have recently been described. Human Th17 and Tc17 cells comprise IL-17-secreting effector T cells that produce little IFN-*γ* [14–17]. These two T cell subsets are CD4^+^ and CD8^+^ T cells [18, 19], respectively, and mounting evidence suggests that Th17 cells, Tc17 cells, and IL-17A (IL-17) have beneficial roles in immune responses to influenza virus infections. Indeed, Wang et al. found that IL-17 mediated B-cell responses and increase survival rates in mice infected with the H5N1 virus [20], and Hamada et al. reported that Tc17 cells protected mice against lethal H1N1 and H3N2 influenza challenge [14]. However, other studies have indicated that IL-17-secreting cells may act as a “double-edged sword,” exacerbating pulmonary inflammation and immunopathology [21–23]. In some studies, H1N1 and H7N9 patients with severe infection showed elevated IL-17A serum levels, and it was proposed that IL-17A might exacerbate lung damage and contribute to the pathogenesis of disease [21, 24, 25]. All of these results highlight the need for further research to clarify the changes in Th17 cells, Tc17 cells, and IL-17A and their roles in influenza virus infection, especially in H7N9 virus infection.

In this study, we investigated changes in Th17 and Tc17 cells in patients with confirmed H7N9 virus infection to clarify the immune status in acute and recovery phases. In addition, we examined the potential roles of Th17 and Tc17 cells and the major sources of IL-17A in H7N9 virus infection.

## 2. Materials and Methods

### 2.1. Patients and Blood Samples

A total of 30 patients were admitted to the First Affiliated Hospital, Zhejiang University School of Medicine, in the fifth wave of human influenza A (H7N9) virus infection from October 2016 to April 2017. In all patients, viral infection was confirmed by reverse transcription polymerase chain reaction (RT-PCR) using clinical samples such as sputum and throat swabs. Medical records for all patients were collected and analyzed. The day of clinical symptom onset was assigned as day 0. The acute phase was defined as day 0 to day 10 from the onset of clinical symptoms, and the recovery phase was defined as day 11 to day 27, as described previously [11]. Peripheral blood samples during these two phases were collected from 20 of the H7N9 patients. Serum was isolated, and blood cells were centrifuged using Ficoll-Paque cell separation medium to collect PBMCs (Cedarlane, Canada). In addition, PBMCs isolated from 20 healthy volunteers and 20 H1N1(2009) patients were obtained as controls. For *in vitro* infection experiments, serum and PBMCs were isolated from 50 healthy volunteers; PBMCs from another 6 healthy volunteers were collected for indirect immunofluorescence assays. This research was approved by the Research Ethics Committee of the First Affiliated Hospital, Zhejiang University School of Medicine, and informed consent was obtained from all patients.

### 2.2. Cell Culture

PBMCs were cultured at 37°C in a humidified atmosphere of 5% CO_2_ and 95% air in RPMI 1640 medium (Gibco, California, USA) supplemented with 10% fetal bovine serum (FBS) (Gibco, California, USA) and penicillin/streptomycin (100 U/mL, 100 *μ*g/mL) (Gibco, California, USA) and then seeded into 24-well tissue plates (Corning Costar, New York, USA) and cultured for the indicated time periods. Madin-Darby canine kidney (MDCK) cells were purchased from ATCC and expanded in Dulbecco's modified Eagle's medium (DMEM; Gibco, California, USA) supplemented with 10% FBS, penicillin (100 U/mL), and streptomycin (100 *μ*g/mL) (Gibco, California, USA) and incubated at 37°C in a humidified atmosphere of 5% CO_2_ and 95% air.

### 2.3. Virus Culture and Infection

The A/Zhejiang/DTID-ZJU01/2013(H7N9) and A/Zhejiang/DTID-ZJU01/2009(H1N1) viruses used in this research were isolated from patients at the First Affiliated Hospital, Zhejiang University School of Medicine. MDCK cell monolayers growing in DMEM containing tolylsulfonyl phenylalanyl chloromethyl ketone-treated (TPCK) trypsin (2 *μ*g/mL) (Sigma, Germany), penicillin (100 U/mL), and streptomycin (100 *μ*g/mL) (Gibco, California, USA) were inoculated with each virus and incubated at 35.5°C in a humidified atmosphere of 5% CO_2_ and 95% air. When 80-90% of the cell monolayer exhibited the cytopathic effect (CPE), the cells were frozen and thawed 3 times, and viruses were harvested. The cultured virus stocks were aliquoted and stored at -80°C until use. For infection, cells were inoculated with the H7N9 or H1N1(2009) virus (20 *μ*L/mL), and infection of human PBMCs was performed as described previously [26]. At 12, 24, and 48 h postinfection, the cells and culture supernatant were collected for analysis. Mock-infected cells were cultured in parallel as controls.

### 2.4. Indirect Immunofluorescence Assay (IFA)

Infected and mock-infected cells were harvested at 24 h postinfection and immediately fixed on slides with 4% paraformaldehyde for 30 min at room temperature. The fixed cells were washed with PBS and permeabilized with 0.5% Triton X-100. The cells were then inoculated with a mouse anti-influenza NP antibody (Abcam, UK) at 4°C overnight, followed by washing with PBS and incubation with FITC-labeled goat anti-mouse IgG (Abcam, UK) for 30 min at room temperature in the dark. DNA staining was performed using DAPI mounting medium. The slides were then washed again and air-dried, and images were captured using a Nikon Eclipse Ti-S fluorescence microscope.

### 2.5. Antibodies and Flow Cytometric Analysis

PBMCs were harvested at the indicated time points postinfection. For the preparation of samples, lymphocyte cell surface markers were stained with the indicated labeled antibodies. To detect intracellular cytokine production by different subsets of T cells, PBMCs were stimulated for 6 h in 24-well plates using 50 ng/mL phorbol 12-myristate 13-acetate (PMA), 1 *μ*g/mL ionomycin, and 500 ng/mL monensin (eBioscience, San Diego, CA, USA). The cells were collected, fixed, and permeabilized using the Fixation/Permeabilization Concentrate (eBioscience, California, USA) according to the manufacturer's instructions. The following flow cytometry antibodies were used for the analyzing cell type and cytokine production: PE-CD4, APC-CD8a, and FITC-IL-17A (BioLegend, California, USA). FITC-, PE- and APC-labeled mouse IgG1·*κ* were utilized as isotype controls (BioLegend, California, USA). Cells were analyzed using a BD FACSCalibur flow cytometer and CellQuest software (BD, California, USA), and the data were evaluated using FlowJo software, version 7.6.5 (TreeStar, San Carlos, CA, USA).

### 2.6. ELISA

For cytokine analysis, serum was separated from the blood of each patient, and culture supernatants were collected at the indicated times after cell stimulation. The level of IL-17A in the serum and supernatant samples were detected using the LEGEND MAX™ Human IL-17A ELISA Kit (BioLegend, San Diego, USA) according to the manufacturer's protocols. Absorbance was measured at 450 nm using the SpectraMax i3x detection system (Molecular Devices, California, USA).

### 2.7. RNA Extraction and Quantitative Real-Time RT-PCR

To detect levels of IL-17A, ROR-*γ*t, and GAPDH mRNA expression, total RNA from human PBMCs was extracted using the QIAGEN RNeasy Mini Kit (QIAGEN, Hilden, Germany), and cDNA was synthesized using a reverse transcription reagent kit (Takara, Dalian, China) according to the manufacturer's protocols. Quantitative real-time RT-PCR was performed using the QuantiFastTM SYBR Green PCR Kit (QIAGEN, Hilden, Germany) with an ABI 7500 instrument (Applied Biosystems, CA, USA). The primer sequences were as follows: IL-17A, forward, 5′-CGGACTGTGATGGTCAACCTGA-3′, reverse, 5′-GCACTTTGCCTCCCAGATCACA-3′; ROR-*γ*t, forward, 5′-CAGAATGACCAGATTGTGCTT-3′, reverse, 5′-TCCATGCCACCGTATTTGC-3′; and GAPDH, forward, 5′-GTCTCCTCTGACTTCAACAGCG-3′, reverse, 5′-ACCACCCTGTTGCTGTAGCCAA-3′. All reactions were carried out in triplicate in the same plate. The relative mRNA level was determined by normalizing the level of the mRNA of interest to that of GAPDH, and the ΔΔCt method was applied to evaluate and compare differential gene expression between samples. The data were analyzed using the ABI 7500 software, v2.0.5 (Applied Biosystems, CA, USA).

### 2.8. Statistical Analysis

*P* values between two groups were calculated using the *t*-test for normally distributed continuous variables or the rank-sum test for nonnormally distributed continuous variables. For more than two groups, *P* values were calculated using one-way analysis of variance (ANOVA) for normally distributed continuous variables and the Kruskal-Wallis test employed for nonnormally distributed continuous variables. When examining categorical variables, *P* values were calculated using the chi-square or Fisher exact test. Correlations between variables were determined by Pearson's correlation coefficient. Data were analyzed using SPSS 19.0 software. For all analyses, *P* < 0.05 was considered statistically significant, and all probabilities were 2-tailed.

## 3. Results

### 3.1. Clinical Characteristics of H7N9 Patients

H7N9, a novel subtype of influenza A virus, is normally confirmed by RT-PCR for viral RNA. Since the first human case was identified in 2013, five major waves of human influenza A (H7N9) virus infections have occurred in Zhejiang Province, China [5]. There were a total of 30 patients with laboratory-confirmed H7N9 infection in the fifth wave of human influenza A (H7N9) virus infection. Of these patients, 16 were male and 14 female. The median age of all patients was 53 years (IQR: 44-64.8). All patients had a history of contact with poultry and developed fever, and the majority also developed cough (90%), sputum (83.3%), and chest distress (63.3%). The most common underlying comorbidities were hypertension (33.3%), diabetes mellitus (10%), malignancy (10%), and chronic liver disease (6.7%) (Table 1). Laboratory results for the two phases of infection revealed significantly higher levels of C-reactive protein (CRP), procalcitonin (PCT), activated partial thromboplastin time (APTT), aspartate aminotransferase (AST), creatinine (Cr), lactate dehydrogenase (LDH), and creatine kinase (CK) in the acute phase than in the recovery phase (Table 2).

### 3.2. Changes in Th17 and Tc17 Cells among PBMCs of H7N9 Patients in the Acute Phase and Recovery Phases

**Figure 1:**
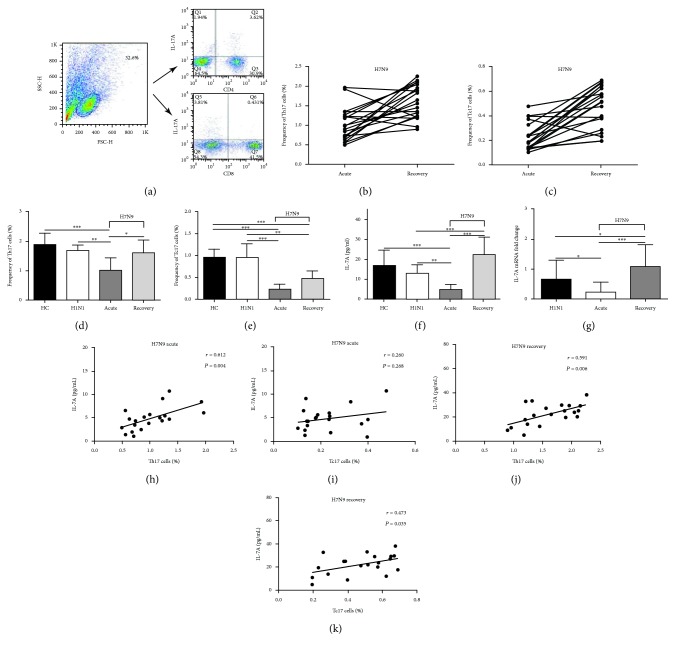
Changes in Th17 cells, Tc17 cells, and IL-17A levels among PBMCs from H7N9 patients in the acute and recovery phases. (a) Isolated PBMCs were gated initially for lymphocytes, and CD4^+^IL-17A^+^ (Th17) and CD8^+^IL-17A^+^ (Tc17) cells were then analyzed. (b, c) The different frequencies of Th17 (b) and Tc17 (c) cells in the acute and recovery phases in different H7N9 patients (*n* = 20). (d, e) Comparison of the proportions of Th17 (d) and Tc17 (e) cells obtained from HC and H1N1(2009) and H7N9 patients in the acute and recovery phases (*n* = 20, respectively). (f) Serum levels of IL-17A in HC and H1N1(2009) and H7N9 patients (*n* = 20, respectively). (g) The fold change in IL-17A mRNA levels in H1N1(2009) and H7N9 patients compared with HC (*n* = 20, respectively). (h, i) Correlation analysis of serum IL-17A levels and Th17 cells (h) and Tc17 cells (i) in H7N9 acute-phase patients. (j, k) Correlation analysis of serum IL-17A levels and Th17 cells (j) and Tc17 cells (k) in H7N9 recovery-phase patients. ^∗^ *P* < 0.05, ^∗∗^ *P* < 0.01, and ^∗∗∗^ *P* < 0.001. HC: healthy controls.

To explore changes in Th17 and Tc17 cells in patients with H7N9 virus infection, PBMC lymphocytes were gated, focusing on Th17 and Tc17 cells by assessing CD4^+^IL-17A^+^ and CD8^+^IL-17A^+^ T cells using flow cytometry (Figure 1(a)). Cells from healthy volunteers (HC) and H1N1(2009) patients were used as controls. In the majority of patients with H7N9 infection, the frequency of Th17 and Tc17 cells increased in the recovery phase compared with the acute phase; the variations observed may be due to individual differences (Figures 1(b) and 1(c)). Th17 cell frequency in H7N9 acute-phase patients was significantly lower than that in HC, H1N1(2009) patients, and H7N9 patients in the recovery phase, and differences among these three groups were not significant (Figure 1(d)). Tc17 cell frequency in acute-phase patients was only slightly lower than that of patients in the recovery phase, but it was apparently lower than that in HC and H1N1(2009) patients (*P* < 0.001). In addition, the difference in Tc17 cell frequency between HC and H1N1(2009) patients was not significant (Figure 1(e)).

### 3.3. Low Serum Protein and mRNA Levels of IL-17A with Reduced Th17 and Tc17 Cell Frequencies in Acute-Phase H7N9 Patients

We next focused on the protein and mRNA levels of IL-17A, which is the effector for Th17 and Tc17 cells. In H7N9 patients, the protein levels of IL-17A in the acute phase were significantly lower than those in HC and H1N1(2009) patients (*P* < 0.001) and were significantly increased in the recovery phase (*P* < 0.001). However, the difference between H1N1(2009) patients and HC was not significant (Figure 1(f)). The changes of IL-17A mRNA were similar to the protein level (Figure 1(g)). In addition, the serum level of IL-17A was positively correlated with the frequency of Th17 and Tc17 cells both in acute and recovered H7N9 patients (Figures 1(h)–1(k)). These results suggest that alterations in IL-17A levels might result from changes in Th17 cell and Tc17 cell frequencies.

### 3.4. H7N9 Virus Effectively Directly Infected Human PBMCs *In Vitro*

**Figure 2:**
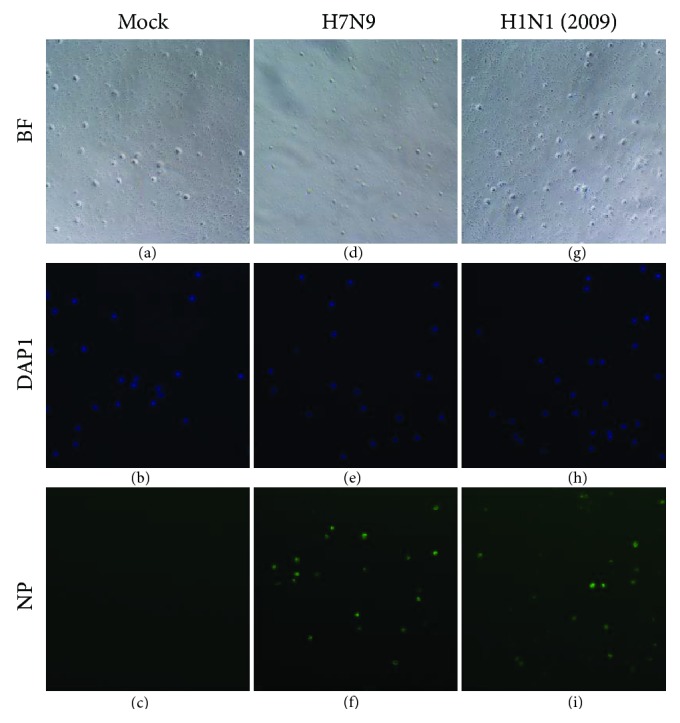
H7N9 virus is able to directly infect human PBMCs *in vitro*. Typical images of immunofluorescent staining of the viral NP protein expressed in mock- (a–c), H7N9- (d–f), and H1N1(2009) virus- (g–i) infected human PBMCs at 24 h postinfection (*n* = 6). Green, NP; blue, DAPI counterstaining of cell nuclei. BF: bright field; DAPI: 2-(4-amidinophenyl)-6-indolecarbamidine dihydrochloride; NP: nucleoprotein. 100x was used.

Isolated PBMCs from the peripheral blood of healthy volunteers were infected with either H7N9 or H1N1(2009) virus *in vitro*; mock- and H1N1(2009)-infected cells were utilized as controls. At 24 h postinfection, PBMCs were fixed, permeabilized, and stained with an anti-NP fluorescence antibody. As shown in Figure 2, abundant viral nucleoprotein- (NP-) positive cells were observed in H7N9- and H1N1(2009)-infected PBMCs but not in mock-infected cells. These data indicate that the H7N9 virus can directly infect human PBMCs *in vitro*.

### 3.5. The Frequencies of Th17 and Tc17 Cells Decreased in Human PBMCs Infected with the H7N9 Virus at Different Time Points Postinfection *In Vitro*

**Figure 3:**
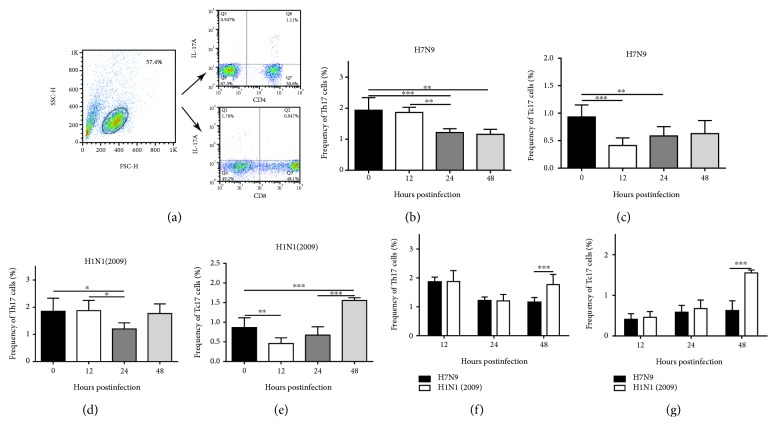
Decreased frequencies of Th17 and Tc17 cells among human PBMCs infected with the H7N9 virus at the indicated time points postinfection *in vitro*. (a) PBMCs isolated from healthy volunteers were infected with the H7N9 or H1N1(2009) virus at the indicated time points after infection; mock-infected and infected cells were gated initially for lymphocytes, and CD4^+^IL-17A^+^ (Th17) and CD8^+^IL-17A^+^ (Tc17) cells were then analyzed. (b, c) Analysis of the proportions of Th17 (b) and Tc17 (c) cells among PBMCs after H7N9 virus infection for 0, 12, 24, and 48 h. (d, e) Analysis of the proportions of Th17 (d) and Tc17 (e) cells among PBMCs after H1N1(2009) virus infection for 0, 12, 24, and 48 h. (f, g) Differences in the frequencies of Th17 (f) and Tc17 (g) cells after H7N9 and H1N1(2009) virus infection for 0, 12, 24, and 48 h. ^∗^ *P* < 0.05, ^∗∗^ *P* < 0.01, and ^∗∗∗^ *P* < 0.001.

To explore the results found for H7N9 patients, a flow cytometry assay was carried out to investigate changes in Th17 and Tc17 cells among PBMCs *in vitro*. Mock- and H1N1(2009) virus-infected cells were used as controls. Among H7N9-infected PBMCs, the frequencies of Th17 and Tc17 cells were decreased at 12 h, 24 h, and 48 h postinfection (Figures 3(b) and 3(c)). Among H1N1(2009)-infected cells, the degree of Th17 and Tc17 cell reduction was smaller, and Tc17 cell frequencies were significantly increased at 48 h postinfection (Figures 3(d) and 3(e)). Figures 3(f) and 3(g) illustrate that the frequencies of Th17 and Tc17 cells infected with the H1N1(2009) virus were higher than in those infected with the H7N9 virus at all time points assessed, and the differences were significant at 48 h postinfection in both cell groups (*P* < 0.001).

### 3.6. Levels of IL-17A Were Reduced in PBMCs Infected with the H7N9 Virus In Vitro

**Figure 4:**
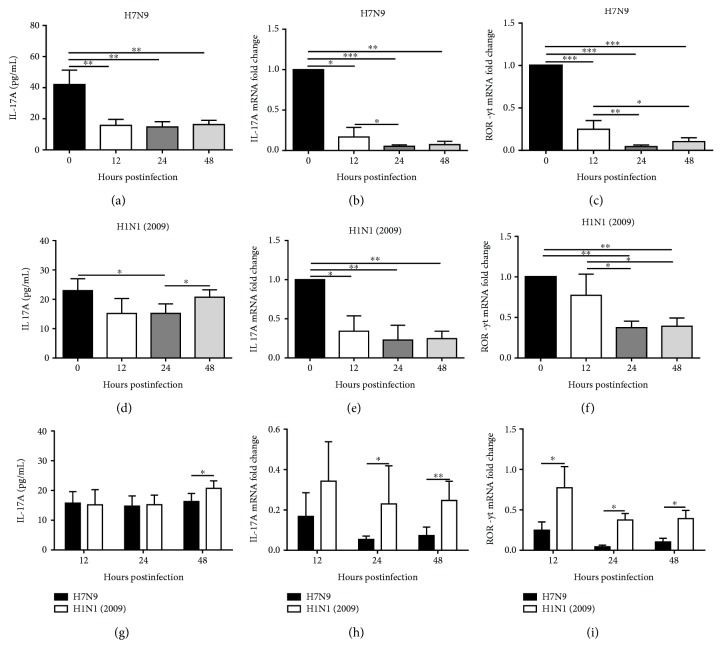
Reduced IL-17A protein and mRNA expression in PBMCs infected with the H7N9 virus *in vitro*. (a–c) Changes in IL-17A protein (a), IL-17A mRNA (b), and ROR-*γ*t mRNA (c) levels after infection with the H7N9 virus at the indicated time points. (d–f) Changes in IL-17A protein (d), IL-17A mRNA (e), and ROR-*γ*t mRNA (f) levels after infection with the H1N1(2009) virus at the indicated time points. (g–i) Comparison of IL-17A protein (g), IL-17A mRNA (h), and ROR-*γ*t mRNA (i) levels after infection with the H7N9 and H1N1(2009) viruses at the indicated time points. ^∗^ *P* < 0.05, ^∗∗^ *P* < 0.01, and ^∗∗∗^ *P* < 0.001.

In our study, ELISA and RT-qPCR were carried out to explore changes in IL-17A protein and mRNA levels. After infection of the H7N9 and H1N1(2009) viruses, both the protein and mRNA levels of IL-17A were decreased at all time points postinfection compared with mock-infected groups (Figures 4(a) and 4(b) and Figures 4(d) and 4(e), respectively). However, the difference in the IL-17A protein level was not significant at 12 h and 48 h postinfection with the H1N1(2009) virus (Figure 4(d)). As depicted in Figures 4(g) and 4(h), H7N9 virus-infected PBMCs showed a significantly lower level of IL-17A protein at 48 h postinfection and less IL-17A mRNA expression at 24 h and 48 h postinfection when compared to H1N1(2009)-infected cells. Moreover, the mRNA level of RAR-related orphan receptor *γ*t (ROR-*γ*t), a transcriptional factor specific to Th17 cells, was also reduced at all time points assessed after infection with both H7N9 and H1N1(2009) viruses (Figures 4(c) and 4(f)). Furthermore, ROR-*γ*t mRNA expression was significantly decreased in H7N9 virus-infected cells compared with cells infected with the H1N1(2009) virus, which was consistent with the changes in IL-17A mRNA (Figure 4(i)).

### 3.7. Th17 Cells Were the Predominant Sources of IL-17A among PBMCs Infected with the H7N9 Virus

**Figure 5:**
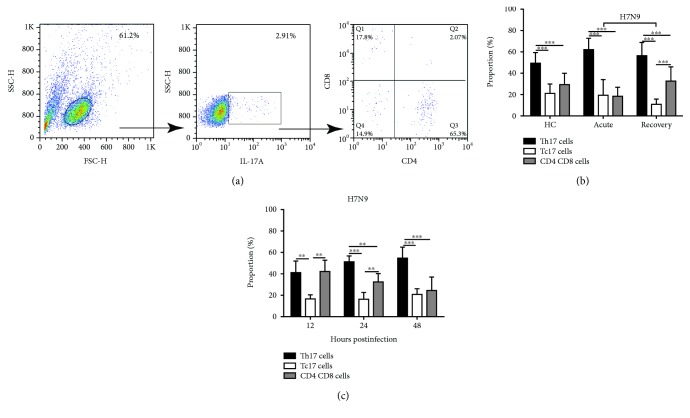
Th17 cells were the principal sources of IL-17A among PBMCs obtained from H7N9 patients and infected with the H7N9 virus *in vitro*. (a) Isolated PBMCs were initially gated for lymphocytes and IL-17A^+^ cells, and the proportions of CD4^+^CD8^−^, CD4^−^CD8^+^, and CD4^−^CD8^−^ cells were then analyzed. (b) The proportions of IL-17A^+^CD4^+^CD8^−^ cells (Th17), IL-17A^+^CD4^−^CD8^+^ cells (Tc17), and IL-17A^+^CD4^−^CD8^−^ cells from HC and H7N9 patients in the acute and recovery phases, respectively (*n* = 20). (c) The proportions of IL-17A^+^CD4^+^CD8^−^ cells (Th17), IL-17A^+^CD4^−^CD8^+^ cells (Tc17), and IL-17A^+^CD4^−^CD8^−^ cells from healthy volunteers infected with the H7N9 virus *in vitro* at the indicated time points. ^∗∗^ *P* < 0.01 and ^∗∗∗^ *P* < 0.001.

To explore the principal source of IL-17A in PBMCs, IL-17A^+^ lymphocytes were gated and the proportions of CD4^+^CD8^−^ (Th17), CD4^−^CD8^+^ (Tc17), and CD4^−^CD8^−^ cells were analyzed (Figure 5(a)). As shown in Figure 5(b), the proportion of Th17 cells was significantly higher than that of Tc17 and CD4^−^CD8^−^ cells among the PBMCs of both acute- and recovery-phase H7N9 patients (*P* < 0.001). In *in vitro* experiments, the proportion of Th17 cells was significantly greater than that of Tc17 cells and CD4^−^CD8^−^ cells at 12 h, 24 h, and 48 h postinfection with the H7N9 virus (Figure 5(c)).

## 4. Discussion

Since emergence of the avian influenza A (H7N9) virus in March 2013, this novel pathogen has posed an ongoing threat to public health. Indeed, this constitutes for the first time that human infection with a low-pathogenicity avian influenza (LPAI) virus has been associated with a high mortality rate [27], which has caused concern worldwide.

This study enrolled 30 patients who had been infected during the fifth wave of human influenza A (H7N9) virus infection. According to the collected medical records, all the patients had a contact history with poultry; most were elderly adults with a median age of 53 years and had underlying diseases/conditions such as hypertension, diabetes mellitus, malignancy, and chronic liver disease. These results are consistent with several previous reports [5, 28–31], demonstrating that the elderly might be at a greater risk of H7N9 virus infection, especially those with underlying diseases/conditions [31, 32]. Additionally, collected laboratory results revealed that CRP, PCT, and serum levels of AST, LDH, and CK were higher in the acute phase than in the recovery phase, suggesting that H7N9 virus infection may lead to severe physiological derangement in acute-phase patients. Our findings are consistent with several previous studies [1, 33–35].

Accumulating evidence demonstrates that Th17 cells and Tc17 cells are IL-17-secreting cells that play important roles in virus infection [36, 37], and it is well established that Th17 and Tc17 cells can aggravate disease in patients with chronic HBV infection [38, 39]. Another study revealed that Th17 cells contribute to the pathogenesis of herpes simplex virus infection [40]. However, it has not been shown conclusively whether these two types of cells are protective or pathogenic in influenza virus infection, and the reported changes in Th17 and Tc17 cells as well as their effector cytokine IL-17A vary among studies. For example, several studies have reported that by downregulating the viral burden in the lung or mediating the B-cell response, IL-17A plays a protective role in H5N1-infected mice and enhances survival rates [20, 41]. Another study revealed that viral load, lung damage, and loss of lung function were all reduced after injecting Tc17 cells into H1N1-infected mice [42]. These findings suggest that IL-17 has a protective role in influenza by promoting virus clearance. Conversely, other studies found increased serum levels of IL-17A in the progression period of influenza infection, reporting that IL-17A might exacerbate lung damage and contribute to disease pathogenesis [21, 43]. In our current study, we limited our focus to changes in Th17 and Tc17 cells in patients with confirmed H7N9 virus infection. Our results showed that the frequency of Th17 and Tc17 cells and the protein and mRNA levels of IL-17A were markedly decreased in acute-phase H7N9 patients and levels were restored in the recovery phase. According to these results, we hypothesize that in the acute phase, decreased levels of Th17 cells, Tc17 cells, and IL-17A might hinder viral clearance and aggravate disease progression. Moreover, a restored cell frequency might be an indicator for predicting recovery from human H7N9 infection. The controversy between our study and those studies that reported increased IL-17A levels is likely related to differences between individuals, study designs, and virus strains. Nonetheless, whether these two types of cells are protective or pathogenic in influenza virus infection needs further study in the future.

Several studies have suggested that influenza virus is able to directly infect B cells and T cells [44, 45]. In our study, IFA results indicated that influenza virus could effectively infect PBMCs *in vitro*, which was in accordance with the previous studies [46, 47] and laid a foundation for ensuing experiments. The results of *in vitro* infection experiments suggested that compared with mock-infected cells, the frequencies of these two groups of cells and IL-17A levels were reduced at all assessed time points postinfection with H7N9, substantiating the findings for acute-phase H7N9 patients. We also detected mRNA levels of ROR-*γ*t, the key transcription factor regulating expression of the Th17 [48] and Tc17 [49] signature cytokine IL-17A. Our results regarding changes in ROR-*γ*t mRNA support those for changes in IL-17A. In addition, when compared with healthy volunteers and H1N1(2009) patients, significantly decreased Th17 and Tc17 cell frequencies and IL-17A levels in acute-phase H7N9 patients were found, though the difference between healthy volunteers and H1N1(2009) patients was not significant. Similar results were found in *in vitro* H7N9 virus-infected cells compared with H1N1(2009)-infected cells. According to these results, we hypothesize that different subtypes of influenza virus might have different impacts on T cell subsets in response to infection [43] and the H7N9 virus might have a more significantly negative impact on Th17 and Tc17 immune responses.

IL-17A is considered to be an important cytokine central to innate and adaptive immune responses to bacterial, fungal, and many viral infections [50, 51]. Accumulating evidence demonstrates that IL-17A is produced by variety of cell types such as *γδ* T cells, NKT cells, NK cells, neutrophils, and eosinophils [52–54]. However, the major source of intracellular IL-17A in PBMCs from H7N9 patients has remained unclear. Our study results show that IL-17A was mainly produced by Th17 cells in PBMCs from H7N9 patients in both the acute and recovery phases as well as at various time points in *in vitro* infection experiments. Additionally, the serum level of IL-17A was positively correlated with the frequency of Th17 cells in H7N9 patients, revealing that changes in IL-17A might predominantly result from changes in Th17 cells among PBMCs from patients infected with H7N9. Furthermore, other studies have reported that *γδ* T cells are the major producers of IL-17 in herpes simplex virus [40] and *Mycobacterium tuberculosis* [52] infection, and in *Schistosoma japonicum* infection, IL-17 is mainly produced by NKT cells [55], suggesting that pathogens might differently affect the phenotype and distribution of IL-17A-producing T cells that act as the main source of intracellular IL-17A cytokine.

There are several limitations to this study. First, our preliminary results were based on a small sample size, which might limit the validity and generalizability of our findings, and further studies with larger sample sizes are required. Second, although we identified decreased frequencies of Th17 cells and Tc17 cells in acute-phase H7N9 patients, further analysis of the molecular and cellular mechanisms for regulating Th17 cells and Tc17 cells in H7N9 infection is needed. Third, animal studies are required to determine whether these two types of cells are protective or pathogenic in H7N9 virus infection.

## 5. Conclusions

In conclusion, our findings indicate that infection with the novel avian influenza A (H7N9) virus can lead to immune disorders in acute-phase patients. Furthermore, the restoration of the frequencies of Th17 and Tc17 cells is a potential biomarker for predicting the recovery of patients with H7N9 infection.

## Figures and Tables

**Table 1:** Clinical features of patients with avian influenza A (H7N9) virus infection.

Variable	H7N9 patients (*n* = 30)
Demographics	
Age, median (IQR) (years)	53 (44-64.8)
Sex, male (%)	16 (53.3)
Contact history with poultry, no. (%)	30 (100)
Clinical signs or symptoms	
Fever, no. (%)	30 (100)
Body temperature, median (IQR) (°C)	40 (38.5-40)
Intolerance of cold, no. (%)	10 (33.3)
Dizziness, no. (%)	5 (16.7)
Headache, no. (%)	2 (6.7)
Muscle ache, no. (%)	5 (16.7)
Weakness, no. (%)	9 (30)
Cough, no. (%)	27 (90)
Sputum, no. (%)	25 (83.3)
Chest distress, no. (%)	19 (63.3)
Hemoptysis, no. (%)	3 (10)
Diarrhea, no. (%)	8 (26.7)
Nausea or vomiting, no. (%)	7 (23.3)
Underlying disease/condition	
Hypertension, no. (%)	10 (33.3)
Diabetes mellitus, no. (%)	3 (10)
COPD, no. (%)	1 (3.3)
Coronary heart disease, no. (%)	1 (3.3)
Malignancy, no. (%)^∗^	3 (10)
Chronic kidney disease, no. (%)	1 (3.3)
Chronic liver disease, no. (%)	2 (6.7)
Hematological disorder, no. (%)	1 (3.3)
Autoimmune disorder, no. (%)	1 (3.3)

Abbreviations: IQR: interquartile range; COPD: chronic obstructive pulmonary disease. ^∗^Malignancy includes tumor and hematologic malignancy.

**Table 2:** Laboratory results for patients with avian influenza A (H7N9) virus infection.

Variable	H7N9 patients (*n* = 30)		*P* value
	Acute phase	Recovery phase	
WBC, median (IQR) (×10^9^ cells/L)	4.8 (3.43-6.98)	5.75 (4-9.65)	0.076
Neutrophils, median (IQR) (×10^9^ cells/L)	4.2 (2.6-6.23)	3.6 (2.3-5.95)	0.561
Lymphocytes, median (IQR) (×10^9^ cells/L)	0.4 (0.3-0.78)	1.2 (0.5-1.78)	**0.001**
CRP, median (IQR) (mg/dL)	92.9 (37.95-141.98)	12.1 (4.4-29.88)	**0.001**
PCT, median (IQR) (ng/mL)	0.31 (0.075-1.61)	0.04 (0.03-0.3)	**0.005**
TT, median (IQR) (s)	17.5 (16-20)	16.8 (15.9-18.1)	**0.046**
APTT, median (IQR) (s)	36 (31.1-44.8)	28.6 (24.3-34.3)	**0.001**
D-D, median (IQR) (ng/mL)	4723.5 (2166.8-8718.8)	2658 (1668-5641)	0.33
ALT, median (IQR) (UI/L)	36.5 (23.5-69.25)	34 (19-57)	0.713
AST, median (IQR) (UI/L)	65.5 (37.5-108.5)	27 (20-55)	**0.001**
TBIL, median (IQR) (*μ*mol/L)	9 (6.25-14)	10 (7-15)	0.353
Cr, median (IQR) (*μ*mol/L)	67 (49.5-109)	55 (46-78)	**0.014**
UA, median (IQR) (*μ*mol/L)	123.5 (91.5-249.3)	155 (117-202)	0.614
LDH, median (IQR) (UI/L)	482.5 (313.5-810.3)	282 (224-358.5)	**0.001**
CK, median (IQR) (UI/L)	208 (83.5-593.8)	46 (22-125.5)	**0.001**
CK-MB, median (IQR) (UI/L)	21 (15-28)	12 (9.5-19)	**0.001**

Continuous variable data are given as the median (IQR). Values in boldface are significant (*P* < 0.05). Abbreviations: IQR: interquartile range; WBC: white blood cells; CRP: C-reactive protein; PCT: procalcitonin; TT: thrombin time; APTT: activated partial thromboplastin time; D-D: D-dimer; ALT: alanine aminotransferase; AST: aspartate aminotransferase; TBIL: total bilirubin; Cr: creatinine; UA: uric acid; LDH: lactate dehydrogenase; CK: creatine kinase; CK-MB: MB isoenzyme of creatine kinase.

## Data Availability

All valuable statistics and data used to support the findings are included within the article, and the results reported in a published article can be found in the publicly archived databases.

## Supplementary Materials

Supplementary Materials Figure S1: very few CD4+IL-17A+IFN-*γ*+ cells and CD8+IL-17A+IFN-*γ*+ cells in patients' and healthy volunteers' PBMCs. Figure S2: very few CD4+IL-17A+IFN-*γ*+ cells and CD8+IL-17A+IFN-*γ*+ cells in PBMCs infected with H7N9 and H1N1(2009) virus in vitro at indicated time points. Figure S3: the frequency of IL-17A+IFN-*γ*- cells and IL-17A+IFN-*γ*+ cells in PBMCs obtained from patients or infected with H7N9 and H1N1(2009) virus in vitro at indicated time points.
