# Supplementary material for: Decreased Frequencies of Th17 and Tc17 Cells in Patients Infected with Avian Influenza A (H7N9) Virus
Source: J Immunol Res. 2019 Apr 2;2019:1418251. doi: 10.1155/2019/1418251 (PMC6466882; doi:10.1155/2019/1418251)
Supplement: Supplementary Materials — Figure S1: very few CD4+IL-17A+IFN-γ+ cells and CD8+IL-17A+IFN-γ+ cells in patients' and healthy volunteers' PBMCs. Figure S2: very few CD4+IL-17A+IFN-γ+ cells and CD8+IL-17A+IFN-γ+ cells in PBMCs infected with H7N9 and H1N1(2009) virus in vitro at indicated time points. Figure S3: the frequency of IL-17A+IFN-γ- cells and IL-17A+IFN-γ+ cells in PBMCs obtained from patients or infected with H7N9 and H1N1(2009) virus in vitro at indicated time points. [file 1418251.f1.pdf]

Figure S1:

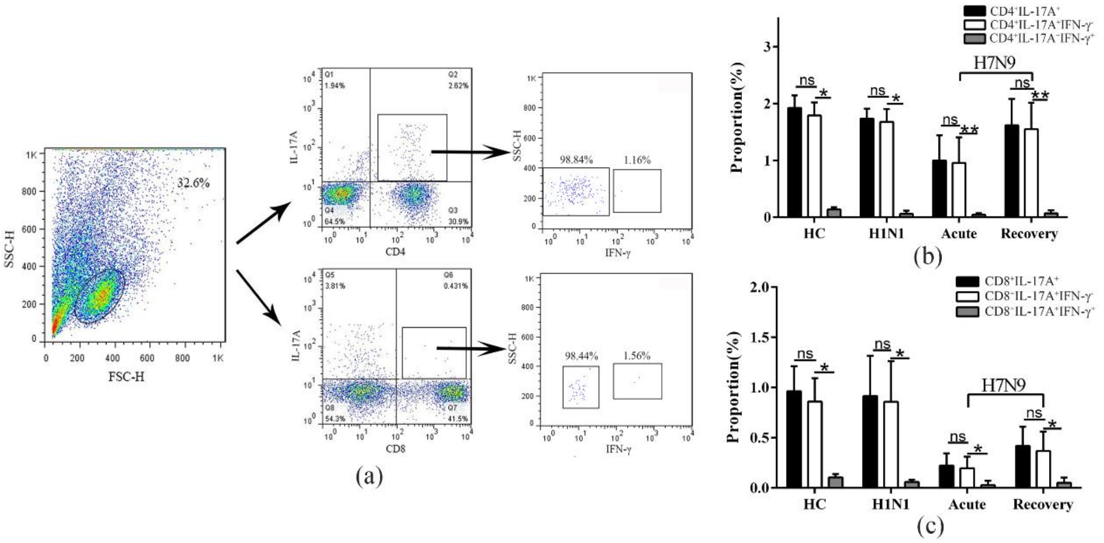

Figure S1: Very few CD4^{+}IL-17A^{+} IFN-γ^{+} cells and CD8^{+}IL-17A^{+}IFN-γ^{+} cells in patients' and healthy volunteers' PBMCs. (a) Isolated PBMCs were gated initially on lymphocytes and then CD4^{+}IL-17A^{+}IFN-γ^{-}, CD4^{+}IL-17A^{+}IFN-γ^{+}, CD8^{+}IL-17A^{+}IFN-γ^{-} and CD8^{+}IL-17A^{+}IFN-γ^{+} were analyzed. (b) The comparison of the frequency of CD4^{+}IL-17A^{+}, CD4^{+}IL-17A^{+}IFN-γ^{-} and CD4^{+}IL-17A^{+}IFN-γ^{+} obtained from HC, H1N1(2009) and H7N9 patients in the acute and recovery phase (n=20, respectively). (c) The comparison of the frequency of CD8^{+}IL-17A^{+}, CD8^{+}IL-17A^{+}IFN-γ^{-} and CD8^{+}IL-17A^{+}IFN-γ^{+} obtained from HC, H1N1(2009) and H7N9 patients in the acute and recovery phase (n=20, respectively). ns, not significant; *, $P<0.05$; **, $P<0.01$.

Figure S2:

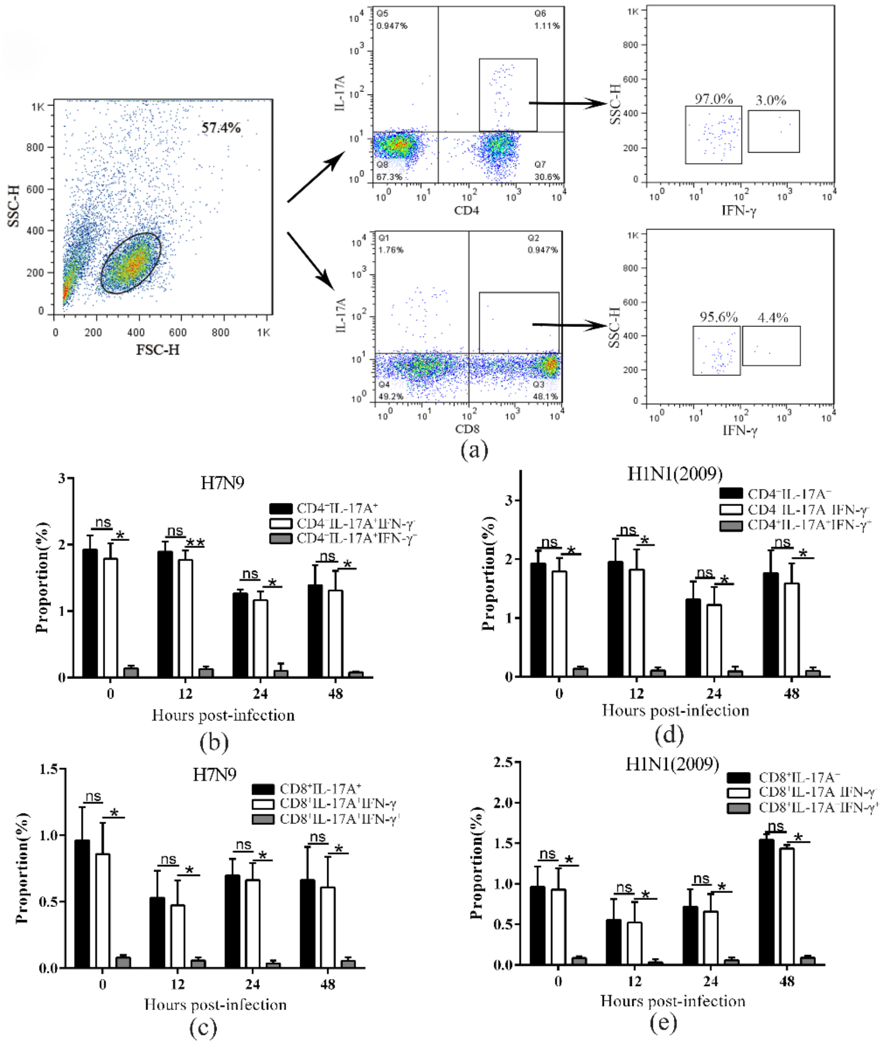

Figure S2: Very few CD4$^+$IL-17A$^+$ IFN-γ$^+$ cells and CD8$^+$IL-17A$^+$IFN-γ$^+$ cells in PBMCs infected with H7N9 and H1N1(2009) virus in vitro at indicated time points. (a) The isolated PBMCs from healthy volunteers were infected with H7N9 and H1N1(2009) virus, at indicated time points after infection, mock-infected and infected cells were gated initially on lymphocytes, then CD4$^+$IL-17A$^+$IFN-γ$^-$, CD4$^+$IL-17A$^+$IFN-γ$^+$, CD8$^+$IL-17A$^+$IFN-γ$^-$ and CD8$^+$IL-17A$^+$IFN-γ$^+$ were analyzed. (b) The comparison of the frequency of CD4$^+$IL-17A$^+$, CD4$^+$IL-17A$^+$IFN-γ$^-$ and CD4$^+$IL-17A$^+$IFN-γ$^+$ by H7N9 virus infection for 0, 12, 24 and 48 h. (c) The comparison of the frequency of CD8$^+$IL-17A$^+$, CD8$^+$IL-17A$^+$IFN-γ$^-$ and CD8$^+$IL-17A$^+$IFN-γ$^+$ by H7N9 virus infection for 0, 12, 24 and 48 h. (d) The comparison of the frequency of CD4$^+$IL-17A$^+$, CD4$^+$IL-17A$^+$IFN-γ$^-$ and CD4$^+$IL-17A$^+$IFN-γ$^+$ by H1N1(2009) virus infection for 0, 12, 24 and 48 h. (e) The comparison of the frequency of CD8$^+$IL-17A$^+$, CD8$^+$IL-17A$^+$IFN-γ$^-$ and CD8$^+$IL-17A$^+$IFN-γ$^+$ by H1N1(2009) virus infection for 0, 12, 24 and 48 h. ns, not significant; *, $P<0.05$; **, $P<0.01$.

Figure S3:

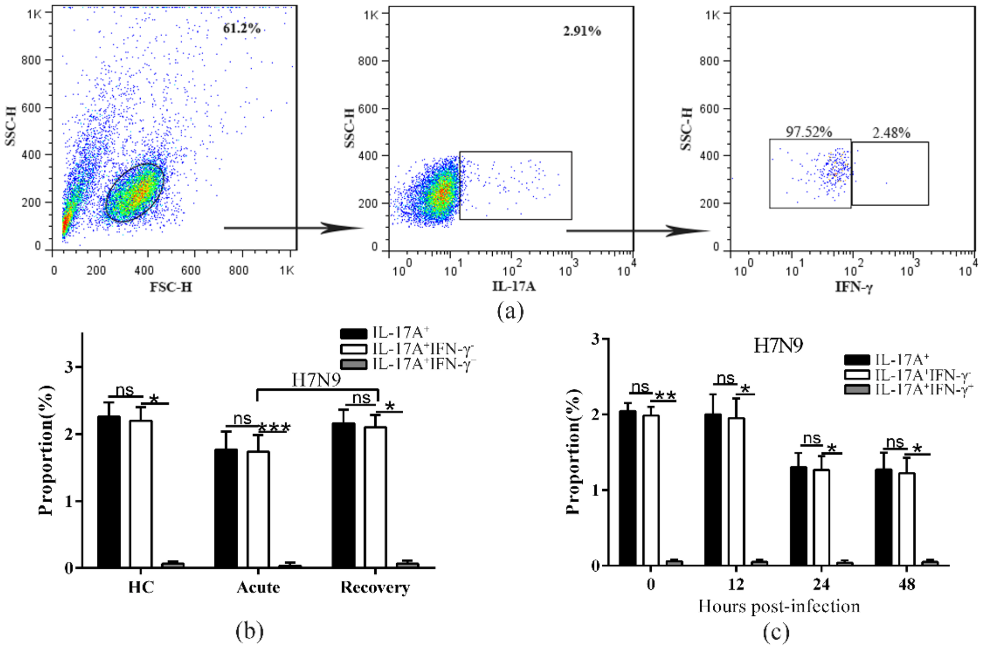

Figure S3: The frequency of IL-17A$^+$IFN-γ$^-$ cells and IL-17A$^+$IFN-γ$^+$ cells in PBMCs obtained from patients or infected with H7N9 and H1N1(2009) virus in vitro at indicated time points. (a) The isolated PBMCs were gated initially on lymphocytes and IL-17A$^+$ cells, then the frequency of IL-17A$^+$IFN-γ$^-$ cells and IL-17A$^+$IFN-γ$^+$ cells were analyzed. (b) The comparison of IL-17A$^+$, IL-17A$^+$IFN-γ$^-$ and IL-17A$^+$IFN-γ$^+$ cells from HC and H7N9 patients in the acute and recovery phase, respectively (n=20). (c) The comparison of IL-17A$^+$, IL-17A$^+$IFN-γ$^-$ and IL-17A$^+$IFN-γ$^+$ cells by H7N9 virus infection for 0, 12, 24 and 48 hours. ns, not significant; *, $P<0.05$; **, $P<0.01$; ***, $P<0.001$.
